# Emodin inhibits respiratory syncytial virus entry by interactions with fusion protein

**DOI:** 10.3389/fmicb.2024.1393511

**Published:** 2024-05-16

**Authors:** Yingcai Xiong, Guangxing Tan, Keyu Tao, Yinghui Zhou, Jun Li, Weiying Ou, Cunsi Shen, Tong Xie, Chao Zhang, Yayi Hou, Jianjian Ji

**Affiliations:** ^1^Wuxi Traditional Chinese Medicine Hospial Afiliated to Nanjing University of Chinese Medicine, Wuxi 214071, China; ^2^School of Medicine, Nanjing University of Chinese Medicine, Nanjing, China; ^3^Jiangsu Key Laboratory of Pediatric Respiratory Disease, Institute of Pediatrics, Nanjing University of Chinese Medicine, Nanjing, China; ^4^The State Key Laboratory of Pharmaceutical Biotechnology, Division of Immunology, Medical School, Nanjing University, Nanjing, China

**Keywords:** respiratory syncytial virus, fusion protein, emodin, entry, small molecule inhibitor

## Abstract

**Introduction:**

Respiratory syncytial virus (RSV) fusion (F) protein is essential for facilitating virus entry into host cells, providing a hopeful path for combating viral diseases. However, F protein inhibitors can rapidly select for viral resistance. Thus, discovering new inhibitors of F-protein is necessary to enrich the RSV drug development pipeline.

**Methods:**

In this study, we screen 25 bioactive compounds from Chinese herbal medicines that exhibit a strong binding to the RSV-F protein using surface plasmon resonance.

**Results:**

After screening, we found emodin could strongly bind to RSV-F protein, and could effectively curb RSV infection. Further investigations certificated that emodin specifically disrupts the attachment and internalization phases of RSV infection by targeting the RSV-F protein. In vivo studies with mice infected with RSV demonstrated that emodin effectively reduces lung pathology. This therapeutic effect is attributed to emodin’s capacity to diminish pro-inflammatory cytokine production and reduce viral load in the lungs.

**Discussion:**

In conclusion, our findings provide initial insights into the mechanism by which emodin counters RSV infection via engagement with the RSV-F protein, establishing it as a viable contender for the development of novel therapeutic agents aimed at RSV.

## 1 Introduction

Respiratory syncytial virus (RSV) causes increased mucus production, inflammation, and airway narrowing, posing a significant threat to children’s health (Haddadin et al., 2021; Qiu et al., 2022). The emergence of RSV outbreaks in China, Europe, and the United States has imposed a significant societal burden (Shao et al., 2022). The management of RSV infection primarily focuses on supportive care, incorporating therapies like bronchodilators, epinephrine, corticosteroids, and hypertonic saline solutions (Smith et al., 2017; Ji et al., 2021). Ribavirin, a nucleoside analog, is the only approved treatment for life-threatening RSV lower respiratory tract infections, and its application is limited due to side effects (Langedijk and Bont, 2023). Small molecule inhibitors are an effective means to treat RSV infection (Langedijk and Bont, 2023), which are not only safe and effective but also cost-effective in combating RSV infections. Therefore, a safe and effective small molecule inhibitor is urgently needed for treatment of RSV infection.

Presently, the majority of antiviral drugs under development for RSV are designed as inhibitors of membrane fusion, specifically targeting the RSV-F protein (Battles et al., 2016; Langedijk and Bont, 2023). The RSV-F protein, a glycoprotein located on the viral envelope’s surface, is essential for facilitating virus entry into host cells (Simoes et al., 2022). Following RSV infection, RSV-F glycoprotein undergoes a series of conformational changes, enabling it to mediate pH-independent membrane fusion (Schlender et al., 2003; Battles and McLellan, 2019). This fusion process allows the release of the viral RNA-nucleocapsid complex into the target cells (Mousa et al., 2017). Fusion inhibitors aim at viral epitopes or cellular receptors, obstructing viral attachment, fusion, and penetration (Olszewska et al., 2011; Battles et al., 2016), which can control infection during the initial phase and limit the subsequent spread of the virus (Battles et al., 2016; Roymans et al., 2017). Therefore, targeting RSV-F protein to interfere with viral entry has emerged as a promising therapeutic strategy (Battles et al., 2016; Roymans et al., 2017). Over the past two decades, numerous structurally distinct inhibitors of the RSV-F protein have been identified and documented (Zheng et al., 2019; Langedijk and Bont, 2023). Numerous inhibitors have effectively advanced to the stage of clinical trials. Ziresovir stands out as a potent, selective, and orally bioavailable inhibitor targeting the RSV-F protein (Zheng et al., 2019). Sisunatovir (RV521), another inhibitor targeting RSV-F, has advanced to Phase IIa trials following a successful human challenge study (DeVincenzo et al., 2020; Cockerill et al., 2021). Nevertheless, the development of most compounds has ceased due to inadequate safety and efficacy demonstrated in clinical evaluations (Langedijk and Bont, 2023). Moreover, candidate RSV inhibitors targeting RSV-F protein face the risk of diminished therapeutic efficacy due to the swift emergence of viral resistance (Stray et al., 2020). Therefore, screening new inhibitors of RSV-F protein is still necessary.

Chinese herbal medicines have long been utilized for treating viral infections, and their bioactive compounds were an important source for the development of antiviral drugs (Xu et al., 2023; Ye et al., 2023). Fox example, polydatin and resveratrol can suppress the replication of various coronaviruses, including HCoV-OC43, SARS-CoV-2 Mpro, and MERS Mpro (Xu et al., 2021); Baicalin blocks RSV infection while diminishing inflammatory cell infiltration and lung damage in murine models (Shi et al., 2016); Resveratrol and emodin can reduce the replication of influenza virus (Kuo et al., 2020). Considering these findings, the screening of antiviral small molecules from Chinese herbal medicines which could be used in the treatment of RSV infection is necessary and holds promise.

In this study, we aimed to screen bioactive compounds from Chinese herbal medicines that exhibit a strong binding to the RSV-F protein using surface plasmon resonance, and the antiviral efficacy against RSV of these small-molecule compounds was validated through both *in vitro* and *in vivo* studies.

## 2 Materials and methods

### 2.1 Reagents

Hesperetin (H107700-1 g), Chrysophanol (D101143-20 mg/A2203416), Polydatin (WXBD1793V) were purchased from Sigma-Aldrich (St. Louis, MO, USA). Emodin (S30748-5 g/T17O11f127680) was purchased from Shanghai yuanye. Apigenin (Q-002-180131) were purchased from Herb purify Co. LTD (Chengdu, China). Rutin (100080–201610) were purchased from National Institutes for Food and Drug Control. Other compounds were purchased from MedChemExpress (Antiviral Compound Library, Cat. No.: HY-L027). RSV-F protein (ser396, #29047, 11,049-V08B/LC17FE2403) was acquired from Sino Biological (Beijing, China). Anti-RSV-F (ab94968) and anti-RSV-G (ab94966) were obtained from Abcam (San Diego, USA). Wuhan University’s Virus Institute (Wuhan, China), provided the Human RSV strain A2 used in this research.

### 2.2 Cells and virus

The human Hep-2 cell line (CRL-9609), and the human lung adenocarcinoma A549 epithelial cell line (CCL-185) were purchased from the ATCC (Manassas, VA, USA). Cells were grown in DMEM (high glucose, Gibco, USA) supplemented with penicillin/streptomycin and 10% heat-inactivated FBS, in a humidified 5% CO2 atmosphere at 37°C. Hep-2 cells were initially used to spread the viruses in a better way or use infection in place of spread. Similarly, after 3 days, the lesions of each pore were observed. When more than 50% of the cells were diseased, the pore was considered to be infected with RSV (or syncytia). The result was calculated by Reed-Muench method. When the virus infects the cells, DMEM containing 2% FBS is used, and the virus that is not bound to the virus is washed twice with PBS.

### 2.3 Cytotoxicity assay

A549 cells were plated at a density of 1 × 10^4^ cells per well in 96-well plates and incubated for a full day. Next, a medium containing emodin at doses ranging from 0 to 100 μM was added to the original media. After a further 24 hours of incubation, each well received the addition of 10 μL of a solution containing 3-(4,5-dimethylthiazol-2-yl) 2,5-diphenyltetrazolium bromide (1 mg/mL). The absorbance of the samples was determined with a microplate reader (TECAN, Infinite 200 PRO, Switzerland) at a wavelength of 570 nm.

### 2.4 Real-time PCR analysis

RNA extraction was carried out using Fast Pure Cell/Tissue Total RNA Isolation Kit (RC101, Vazyme, Nanjing, China) and cDNAs were synthesized with a HiScript II Q RT SuperMix for qPCR with gDNA wiper (R223, Vazyme, Nanjing, China). Real-time PCR analysis was performed using ChamQ SYBR Color Qpcr Master Mix (Q431, Vazyme, Nanjing, China). Primer sequences are detailed as follows (forward and revers 5′-3′): human GAPDH: GCACCGTCAAGG CTGAGAAC and ATGGTGGTGAAGACGCCAGT, mouse GAPDH: AACGACCCCTTCATTGAC and TCCACGACATACTCAGCAC, RSV-A (N region): CATCCAGCAAATACACCATCCA and TTCTG CACATCATAATTAGGATATCAA, RSV-A (G region): CGGCAA ACCACAAAGTCACA and TTCTTGATCTGGCTTGTTGCA, RSV-A (F region): AACAGATGTAAGCAGCTCCGTTATC and GATTTTTA TTGGATGCTGTACATTT, Mouse IL-1β: GCAACTGTTCCTGA ACTCAACT and ATCTTTTGGGGTCCGTCAACT, Mouse TNF-α: CCCTCACACTCAGATCATCTTCT and GCTACGACGTGGGCTACAG.

### 2.5 Immunofluorescence confocal microscopy

Cells were stabilized using 4% paraformaldehyde at ambient temperature for 15 min, followed by staining with a primary antibody targeting the RSV-G protein (1:1000, 94,966, Abcam, USA), Next, they were incubated with a secondary anti-mouse antibody (1:1000, SA00013, Proteintech, China) labeled with Alexa Fluor 488. After coating the samples with an anti-fluorescence quencher (P0131, Beyotime, China) to seal them, they were stained with DAPI (C1005, Beyotime, China) and examined using a confocal laser scanning microscope (TCS SP5; Lieca, Germany).

### 2.6 Surface plasmon resonance

Reichert 4SPR system was used to evaluate its binding affinity with RSV-F protein by SPR technique. Initially, RSV-F protein was anchored onto the sensor chip by amino coupling to achieve an optimal quantity. The chip was exposed to varying concentrations of emodin for analysis of association and dissociation rates. Data collection occurred at 25°C using PBST running buffer (8 mM Na2HPO4, 136 mM NaCl, 2 mM KH2PO4, 2.6 mM KCl, and 0.05% Tween 20, pH 7.4) with 1% DMSO. Data analysis was conducted using TraceDrawer software, and GraphPad Prism 9 was utilized for result visualization.

### 2.7 MicroScale thermophoresis

RSV-F protein was labeled using the Monolith His-Tag Labeling Kit (MO-L018, Nano Temper). The labeling procedure is described in detail in the operating manual of the kit and is outlined below. Firstly, the affinity and labeling efficiency between the dye and His-tagged proteins were first detected, and the reaction buffer system was PBST. According to the affinity between dye and protein obtained in the previous step, different steps were selected to label proteins. After protein labeling, RSV-F protein was added to 1–16 tubes at a final concentration of 50 nM. A 2-fold dilution of emodin was subsequently added simultaneously to each tube. The highest concentration of emodin was 100 μM. The samples were centrifuged at 12000 g for 10 min at 4°C, and the supernatant was aspirated using a capillary tube (MO-K022-SP, NanoTemper). The binding affinity was detected with Monolith NT.115 (NanoTemper Technologies, Munich, Germany).

### 2.8 Molecular docking

The structure files of Emodin (PubChem CID: 3220) and RSV-F (HRSVgp08, PDBID: 4CCF) protein were obtained from PubChem and Protein Date Bank (PDB). The RSV-F protein underwent structural preparation via the Maestro tool within the Schrodinger suite, entailing the excision of water molecules, the annexation of hydrogen atoms, and the amendment of structural aberrations. Subsequently, the entirety of the RSV-F protein was designated as the docking target. Energy minimization of the Emodin molecule was executed to secure its lowest energy conformation. The Ligand Docking approach was employed for molecular docking, accentuating the ligand’s interactions with the target protein. Post-docking, the outcomes were archived in the mdb file format, discovery and PyMOL was utilized for the visual dissection of the results.

### 2.9 Time of addition

According to the process of RSV infection, the experiment was divided into pre-binding, binding and post-binding stages. At different stages of RSV infection, emodin was added to determine the action stage of emodin. Free compounds and viruses were washed off using PBS at each stage turnover. In addition, the virus can only adsorb but not enter the membrane fusion at 4°C. We prevented the virus from fusing with the cell membrane by keeping the cell at 4°C, and after the virus adsorbed, we raised the temperature to 37°C, at which point the virus began to fuse with the cell membrane. The membrane fusion of virus and cells was controlled by controlling the temperature, and then emodin was added at different stages to determine the effect of emodin on the membrane fusion of virus.

### 2.10 *In vivo* murine model infection using RSV

Female BALB/c mice, aged 6 weeks and weighing 18-20 g, were procured from Nanjing Qing zilan Technology Co., Ltd., China, for experimental use. RSV (1.8 × 10^6^ PFU/mouse) was intranasally inoculated. Emodin (10, 40, 100 mg/kg/day) were dissolved in an aqueous solution containing 1% DMSO or distilled water with DMSO were given to the mice by nasal drops for 3 days (n = 5). All experimental protocols were conducted following the National Institutes of Health Guidelines for the Care and Use of Laboratory Animals and received approval from the Animal Ethics Committee at Nanjing University of Chinese Medicine (Permission Number: 202309A017).

### 2.11 Statistics and reproducibility

Unless otherwise stated, all the results are given as the means ± SEM of three separate studies. For comparisons among multiple groups, one-way analysis of variance (ANOVA) was utilized, followed by *post hoc t*-tests with Bonferroni correction. The GraphPad Prism 9 software for Windows (GraphPad Software, San Diego, CA, USA) was used to do all of the computations. *p* values of less than 0.05 were regarded as statistically significant.

## 3 Results

### 3.1 Emodin can bind to RSV fusion protein

**Figure 1:**
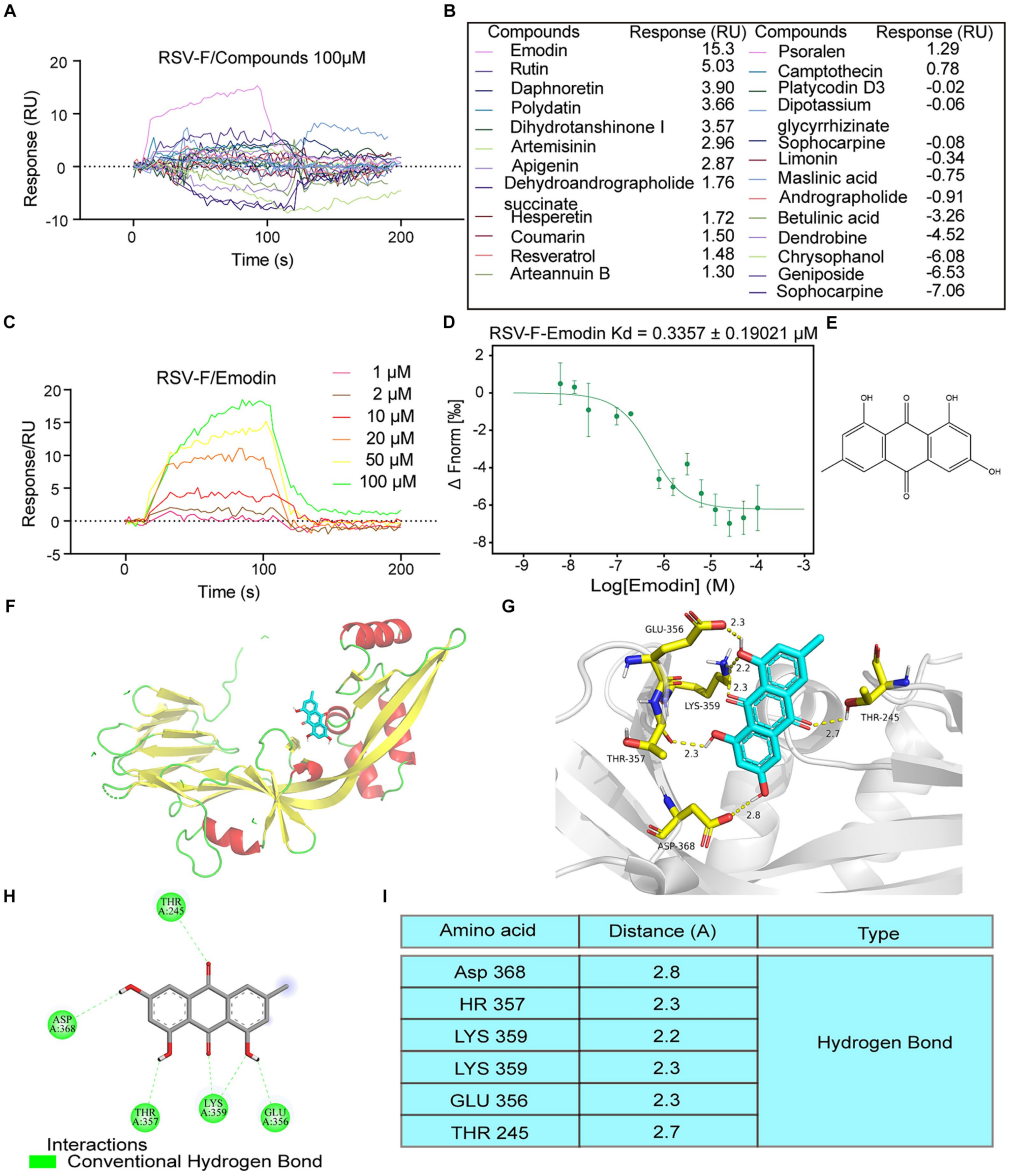
Intermolecular interactions between different molecules and RSV F proteins. **(A,B)** Interaction of the compounds with RSV-F protein at concentration of 100 μM. **(C)** 1, 2, 10, 20, 50, 100 μM concentrations of emodin interact with RSV-F protein by SPR. **(D)** Verified the interaction between emodin and RSV-F protein by MicroScale Thermophoresis. **(E)** Chemical structure of emodin. **(F,G)** Views for emodin bound to RSV-F. Each RSV-F protomer is again shown in a different color corresponding to the colors. Emodin is shown as ball-and-stick representation with colors of atoms corresponding to the colors in a. d 2D ligand-interaction diagram generated in Molecular Operating Environment. **(H)** The binding amino acids between compounds and F protein. **(I)** The distances between emodin and the interacting amino acid residues.

Traditional Chinese Medicine (TCM) has a profound historical background in the treatment of infectious diseases (Huang et al., 2021). For this study, we selected 25 small compounds from TCM, which has been reported to possess antiviral effects. However, whether they could inhibit RSV infection or the mechanism of anti-RSV infection is unclear. Some compounds with well anti-RSV effects and relatively clear mechanisms are not included in this list, such as quercetin and baicalin. Then, we evaluate whether these bioactive compounds can bind to the RSV-F protein using surface plasmon resonance (SPR) experiments. The RSV fusion (F) protein was anchored onto the sensor chip surface to achieve the desired quantity. Then bioactive compounds at 100 μM was injected over the sensor chip surface for association analysis, followed by dissociation analysis. The results showed that emodin and rutin could bind to RSV-F protein at 100 μM, however other compounds showed no interaction (Figures 1A,B). Emodin displayed stronger binding ability to RSV-F protein than rutin (Figures 1A,B). Therefore, we focus on emodin in the next experiments. Subsequent experiments revealed that the interaction between emodin and the RSV-F protein demonstrated a dose-dependent relationship, with the minimal effective concentration being 10 μM (Figure 1C). Also, microscale thermophoresis (MST) experiment was used to assess the binding interaction between emodin and the RSV-F protein. The MST analysis provided further support by confirming the dose-dependent binding behavior of emodin to the RSV-F protein (Figure 1D). Collectively, these analyses suggest that emodin can strongly bind to RSV-F protein.

Next, we explored the binding sites of emodin and RSV-F using molecular modeling. Figure 1E provides a visual representation of its molecular structure of emodin. The binding sites of emodin to RSV-F protein was illustrated in Figures 1F,G, indicating where emodin interacted with RSV-F protein. The results showed emodin could bind to various amino acids (LYS359, THR357, GLU356, THR245, and ASP368) on the RSV-F protein in the form of hydrogen bonds (Figures 1H,I). The analyze data also displays the distances between emodin and the interacting amino acid residues, providing a visual representation of the spatial proximity between emodin and the specific amino acids involved in the binding sites.

### 3.2 Emodin inhibits RSV infection *in vitro*

**Figure 2:**
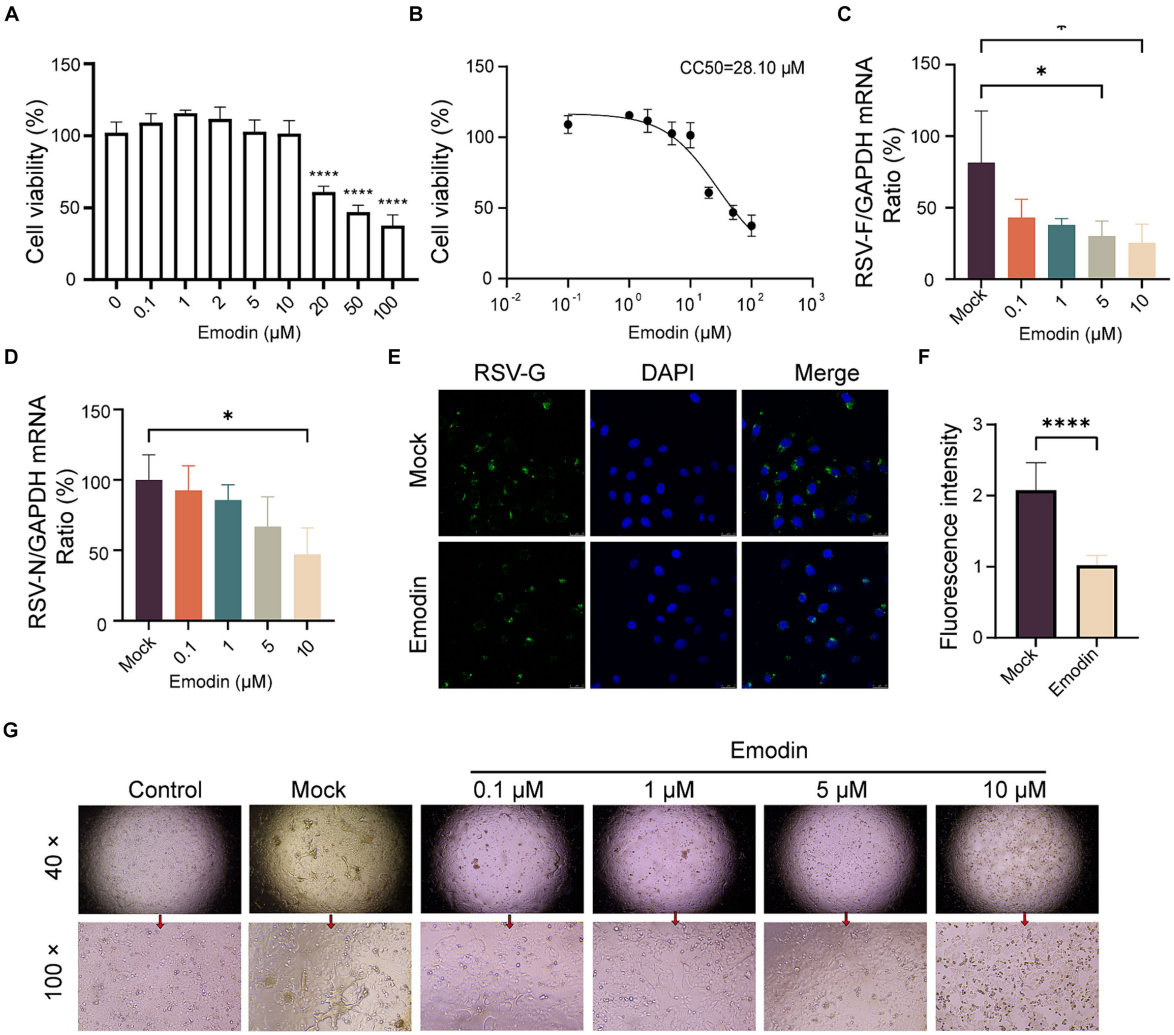
Antiviral activity of emodin against RSV. **(A,B)** The CCK8 assay was used to determine the safe administration concentrations of the emodin, and the data from the experiments were plotted to extrapolate the CC50 values (*n* = 6). **(C,D)** RSV N and F protein expression was detected to determine RSV infection by RT-qPCR, (*n* = 3). **(E,F)** The detection of RSV-G protein after treatment with emodin, and the mean fluorescence intensity was calculated (*n* = 9). **(G)** RSV infection induced cytopathic effect in A549 cells was evaluated. A549 cells were treated with emodin in combination with the RSV mixture for 1 h, followed by removal of the mixture using PBS and addition of DMEM containing 2% FBS. The culture was continued for 3 days to observe the formation of syncytia. Data are expressed as mean ± SEM. ^∗^*p* < 0.05, ^∗∗^*p* < 0.01, ^∗∗∗^*p* < 0.001, ^∗∗∗∗^*p* < 0.0001. ns, not significant.

Interfering with the RSV-F protein can prevent viral entry (Schlender et al., 2003). We concluded that emodin might inhibit RSV infection through targeting RSV-F protein. Initially, we evaluated the cytotoxic effects of emodin under our experimental setup by exposing A549 cells to diverse concentrations of emodin over a 24-h period. To determine the cytotoxic effects, we employed a CCK8 assay. The experimental data indicate that emodin did not exhibit any discernible cytotoxic effects at concentrations ranging from 0 to 10 μM (Figure 2A). (Figure 2A). The CC50 value for emodin was 28.10 uM (Figure 2B).

We then assess the antiviral effects of emodin. Initially, A549 cell monolayers were exposed to emodin, meanwhile by RSV infection for a duration of 24 h. Using RT-qPCR to detects the expression of RSV-F and RSV-N genes. It was showed emodin, resulted in a notable decrease in RSV-F gene expression (Figure 2C). The expression of RSV-N gene was also decreased after emodin treatment at 10 μM (Figure 2D). In addition, the immunofluorescence results also proved the antiviral effect of emodin (Figures 2E,F). Multinucleated giant cells may develop as a result of cell fusion triggered by RSV infection. Treatment with emodin can effectively reduce this cell fusion (Figure 2G). In summary, these results collectively demonstrate that emodin inhibits RSV infection in a dose-dependent manner.

### 3.3 Emodin interferes with RSV attachment and internalization through influencing RSV-F protein rather than the receptors on host cells

**Figure 3:**
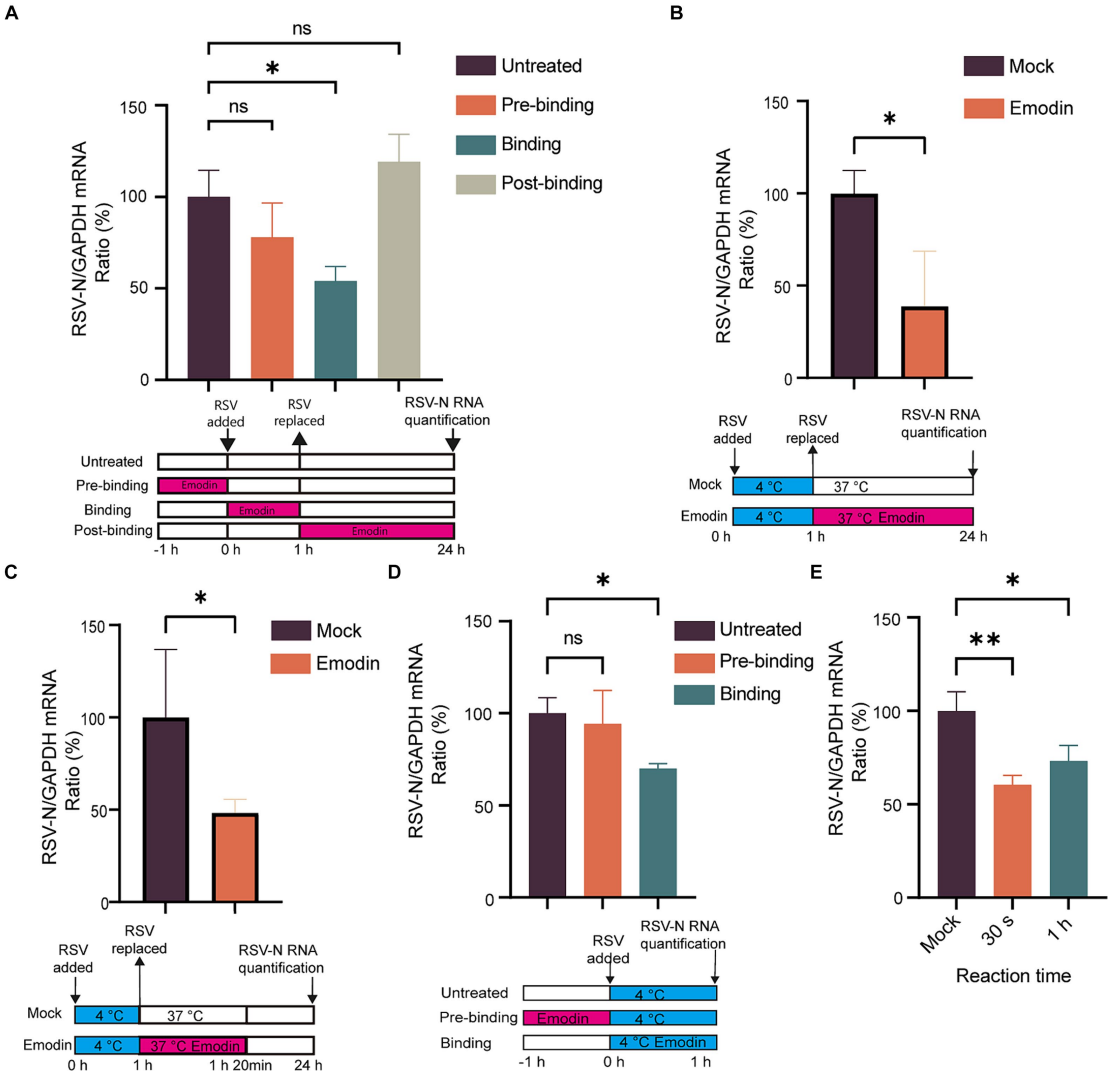
Attachment and internalization assay for emodin. **(A)** Anti-RSV activity of emodin in A549 cells. A549 cells were infected with RSV (1 MOI) and treated with emodin (10 μM) at the pre-entry phase, entry phase, replication phase, followed by culture for 24 h. The culture condition was illustrated bellow. RSV-N protein expression was detected to determine RSV infection by RT-qPCR (*n* = 3). **(B)** Schematic of the attachment procedure (down). A549 cells were infected with RSV in the presence or absence of emodin at the binding phase, and then cultured for 24 h (*n* = 3). Viral load was determined by RT-qPCR (up). **(C)** Schematic of the internalization procedure (down). RSV (1 MOI) was added to A549 cells, which were then incubated at 4°C for 1 h. After absorption, the medium containing the virus was removed, the emodin at 10 uM was added to the cells, and the plates were incubated at 37°C for 20 min. After washing the cells with phosphate-buffered saline (PBS), the cells were cultured for 24 h (*n* = 3). **(D)** Schematic of the attachment procedure (down). A549 cells were pretreated with emodin for a duration of 1 h prior to RSV infection. Subsequently, the viral load was measured 1 h after RSV infection to evaluate the impact of emodin on viral load. Viral RNA levels in the cells were measured by RT-qPCR (*n* = 3). **(E)** Emodin directly inhibits the RSV infectivity. High-concentration RSV was incubated with emodin (10 μM) for 0.5 or 60 min. A549 cells were inoculated with the treated RSV (1 MOI) and cultured for 24 h (emodin concentration 0.01 μM). Levels of viral RNA in the cells were measured by QRT-PCR (*n* = 3). Data are expressed as mean ± SD. ^∗^*p* < 0.05, ^∗∗^*p* < 0.01, ^∗∗∗^*p* < 0.001, ^∗∗∗∗^*p* < 0.0001. ns, not significant.

Numerous studies have highlighted the crucial role of RSV-F protein in viral attachment and membrane fusion (Battles et al., 2016). Viral attachment and membrane fusion can be summarized as the process of viral entry into cells. Considering that we conducted time-of-addition assays to investigate whether emodin could inhibit RSV entry. The viral life cycle including attachment, membrane fusion, RNA release, replication, assembly and budding. Next, we focused on identifying the phases within the viral life cycle where emodin exerts its inhibitory effects. As depicted in Figure 3A, the inclusion of emodin during the virus entry phase led to a substantial decrease in RSV infection (Figure 3A). Nevertheless, its effectiveness was reduced when administered during the post-entry or pre-entry phases, suggesting that emodin primarily exerts its influence during the entry stages of RSV infection.

Viral entry into host cells occurs through attachment to the cell surface and subsequent membrane fusion (Battles et al., 2016). Therefore, we conducted assays to investigate the effect of emodin on RSV attachment and fusion. The virus can adsorb to the surface of host cells at low temperature, but cannot fuse with the host cell membrane. To explore the effect of emodin on RSV attachment, cells were infected with a mixture of RSV and emodin at 4°C. At this time, the virus can adsorb to the cell surface but cannot undergo membrane fusion. After 1 h, the mixture was washed off by PBS. The RSV-N gene expression was measured at 24 h. The data showed that emodin treatment led to a reduction of RSV-N gene expression. It is indicated that emodin could prevents the virus from attaching to the cell (Figure 3B). To explore the effect of emodin on RSV internalization, A549 cells were initially incubated at 4°C for 1 h to facilitate virus attachment. Subsequently, the temperature was shifted to 37°C for a duration of 20 min. Allowing the virus to undergo membrane fusion. The cells were treated with emodin at the same time. The viral load was then evaluated at 24 h by measuring the expression of RSV-N gene. The results indicated that emodin significantly reduced the viral load during the RSV membrane fusion stage (Figure 3C).

The above results indicate that emodin can bind to RSV-F protein and inhibit RSV adsorption to cells and carry out membrane fusion. This effect may be caused by emodin interacting with RSV-F, or by influencing viral adsorption and membrane fusion receptors expressed by cells. Then, we next investigated whether emodin could influence the receptors on host cells. A549 cells were subjected to pre-treatment with emodin 1 h prior to RSV infection, and in other group, A549 cells were treated with emodin in the RSV binding phase for 1 h at 4°C (Figure 3D). Viral load was then measured 1 h after RSV infection. We observed a significant decrease in viral RNA level when emodin treatment at the binding stage, and however emodin pre-treatment did not exert any influence on viral load (Figure 3D). This result suggested emodin had a direct effect on RSV-F protein rather than the host cells. To further examine the potential immediate and prolonged impacts of emodin on virions, we subjected a concentrated RSV solution to emodin incubation for durations of both 30 s and 1 h. After emodin-treated the virus infected A549 cells at a MOI of 1 for 24 h (Note: When the virus infects the cells, the emodin concentration is less than 0.01 μM, which is no longer able to inhibit RSV infection). The result of this experiment showed a significant decrease in viral gene expressions when the virions were treated by emodin for both 30 s and 1 h (Figure 3E). It indicating that emodin possessed direct inhibitory effect on RSV its self. To summarize these results, emodin influences RSV entry into host cells by interacting with RSV-F protein.

### 3.4 Emodin demonstrates antiviral activity and offers protection against RSV-induced lung pathology *in vivo*

We assessed the antiviral efficacy of emodin through the utilization of a mouse model. The preceding set of investigations has suggested that emodin exhibits a notably low level of bioavailability (Dong et al., 2016). Hence, mice that were infected with RSV received treatment through intranasal administration. Emodin or water was intranasal administrated for 3 days. Mice were euthanized on day 4, and their lung tissues were extracted for the assessment of viral production and pathological analysis.

**Figure 4:**
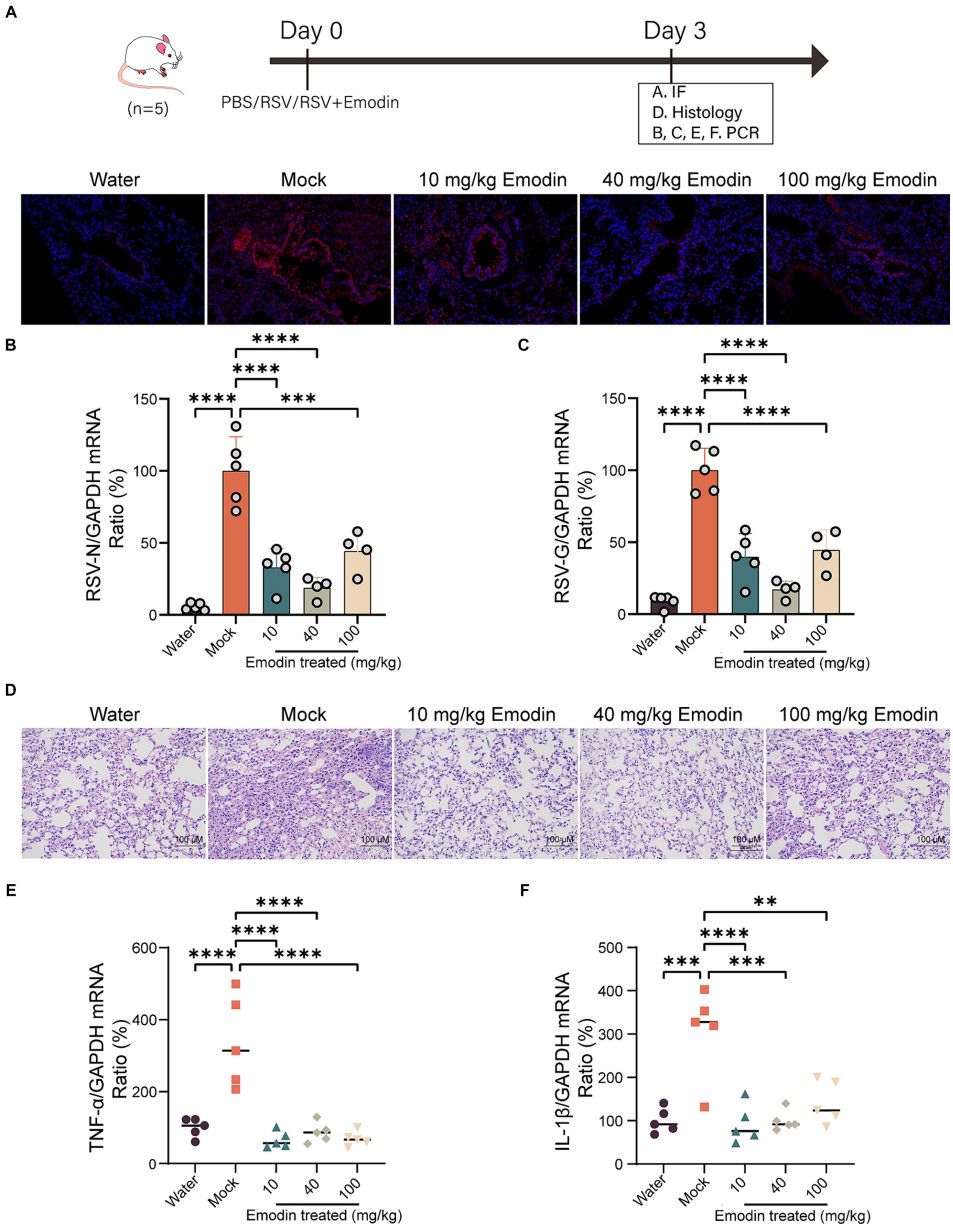
Emodin reduced RSV infection *in vivo*. **(A)** RSV-F protein staining for evaluating the viral load in the lungs of mice using immunofluorescence (*n* = 3). Red: RSV-F protein; Blue: DAPI. **(B,C)** The viral load in the lungs of mice was measured by viral RNA (N and G) expression in relation to GAPDH (*n* = 5). **(D)** Representative hematoxylin and eosin (H&E)-stained lung tissue from mice showed histologic differences following 3 days of infection (*n* = 3). **(E,F)** Expression of inflammation molecules in lungs of all mice were determined by RT-qPCR 3 days after infection (*n* = 5). Data are represented as mean ± SEM. ^∗∗^*p* < 0.01, ^∗∗∗^*p* < 0.001, ^∗∗∗∗^*p* < 0.0001. ns, not significant.

As shown in Figure 4A, the viral titers in lung tissues treated with emodin were lower compared to the vehicle-treated group. Emodin also reduced the expression of RSV genes in the lungs (Figures 4B,C).

We also conducted a detailed examination of lung inflammation following emodin treatment. Histological studies have shown that lung damage caused by RSV infection and the accumulation of inflammatory cells are clearly visible in the lung tissue Nonetheless, emodin treatment effectively mitigated these effects (Figure 4D). Meanwhile, after emodin treatment, the expression levels of tumor necrosis factor α (TNF-α) and interleukin-1β (IL-1β) in lung tissues were decreased (Figures 4E,F).

## 4 Discussion

The field of RSV therapy has significantly increased its therapeutic potential since the discovery of the pre-F conformation of the RSV-F protein (Mousa et al., 2017; Langedijk and Bont, 2023). Currently, the world’s first vaccines to prevent RSV among older adults, have recently been approved (Langedijk and Bont, 2023). However, during clinical trials, concerns have been raised regarding the potential risk of the pre-F vaccine inducing Guillain-Barré Syndrome (Langedijk and Bont, 2023). The U.S. Food and Drug Administration (FDA) has authorized the use of the RSV monoclonal antibody Nirsevimab-alip for the prevention of lower respiratory tract disease caused by RSV in infants and children under the age of two (Harris, 2023). However, the dissemination of Nirsevimab has been hindered by its high cost and bureaucratic barriers within the vaccine distribution system for children under medical subsidy (Eisenstein, 2023). Recently, the FDA has approved the first maternal RSV vaccine, Abrysvo, aimed at preventing severe RSV diseases in newborns to infants up to six months old. However, it may increase preterm births (Eisenstein, 2023). Therefore, a safe and effective small molecule inhibitor is urgently needed for treatment of RSV infection.

TCM, such as *Polygonum cuspidatum*, has been observed to exert inhibitory activities against several viruses. Several studies have conducted assays to screen natural inhibitors from for defense against influenza (Wang et al., 2023), SARS-CoV-2 Wild-Type, Omicron Pseudotyped viruses (Lin et al., 2022), dengue virus (Kuo et al., 2020). In this study, we selected 25 compounds and evaluated their binding ability to RSV-F proteins. Our results showed that emodin and rutin can bind to F protein, and emodin has stronger binding ability to F protein. Molecular analysis was used to predict the binding sites between emodin and RSV-F protein. However, the specific binding sites require further confirmation through techniques such as X-ray diffraction for a more detailed analysis.

Emodin possessed broad-spectrum antiviral ability, which can inhibit more than 10 viruses replications (Shao et al., 2022). It is generally believed that antiviral effects are mainly achieved by affecting host cell signaling pathways associated with chronic inflammation and lung damage (Shao et al., 2022). Previous study also reported emodin could limit RSV infection *in vitro* (Liu et al., 2015) however the mechanism is unknown. In this study, our study also indicates that treatment with emodin can significantly reduce the infection of respiratory syncytial virus *in vivo* and *in vitro*, which is consistent with previous study (Liu et al., 2015). Emodin treatment also ameliorated pulmonary pathology and decreased the expression of pro-inflammatory genes *in vivo*. In mechanism, emodin can interfere with RSV attachment and internalization by directly target RSV-F protein. Moreover, numerous reports have demonstrated that emodin possesses significant anti-inflammatory activity (Hu et al., 2023), suggesting that the therapeutic mechanism of emodin in RSV infection includes anti-inflammation effects. Our data provide evidences that emodin may be as therapeutics to against RSV infections.

Despite its promising antiviral activity *in vivo* and *in vitro*, emodin has some problems such as side effects and poor bioavailability of emodin (Dong et al., 2020). Although we observed that emodin exhibited efficacy at a concentration of 10 uM, we were also aware of its CC50 value of 28.10 uM, which was only a threefold difference. And the liver is considered one of the primary target organs in the toxicological studies of emodin, and nephrotoxicity of emodin is also discovered (Dong et al., 2020). It has been reported that emodin may possess reproductive toxicity and genotoxicity characteristics (Dong et al., 2020). Furthermore, the significant first-pass effect of emodin in the liver and intestines plays a crucial role in its limited oral bioavailability (Dong et al., 2016). Hence, it is imperative to enhance bioavailability in order to reduce the administered dose and consequently mitigate drug toxicity. Moreover, modification of emodin to improve its water solubility is also a reasonable way to improve its bioavailability. In recent years, numerous studies have focused on addressing these limitations, and substantial progress has been made. These approaches involve modifications to physical or chemical properties, along with the incorporation of solvents or surfactants. For more detailed information, please refer to our previous article (Shao et al., 2022).

In this study, emodin was delivered via intranasal administration, a route that may circumvent challenges associated with suboptimal oral bioavailability. Intranasal delivery offers a pronounced benefit for the prophylaxis and treatment of respiratory tract infections, allowing direct targeting of the site of viral invasion. However, this method is not without limitations. A primary obstacle is ensuring efficient dispersion of the therapeutic agent across the expansive mucosal surface of the airway epithelium, especially considering the constraints imposed by the limited volume of application. Furthermore, there exists an apprehension that intranasal delivery of liquids could inadvertently enhance viral spread and exacerbate pulmonary infections. To address these challenges, further refinements in delivery strategies, such as aerosolized inhalation, and the formulation of drugs tailored for pulmonary administration are warranted.

## 5 Conclusion

In conclusion, emodin can effectively bind to the RSV-F protein and impeded RSV attachment and internalization. Treatments with emodin significantly ameliorated pulmonary pathologies in RSV infected mice. This study illustrates that the emodin may be a new antiviral strategy in RSV infection. In subsequent studies, we advocate for the optimization of emodin’s antiviral efficacy. Strategies may involve targeted structural alterations to improve its affinity for the RSV-F protein, as well as utilizing nanotechnology for encapsulation to attenuate emodin’s toxicity.

## Data availability statement

The original contributions presented in the study are included in the article/Supplementary material, further inquiries can be directed to the corresponding authors.

## Ethics statement

The animal study was approved by the Ethics Committee of the Laboratory Animal Center at Nanjing University of Chinese Medicine, China. The study was conducted in accordance with the local legislation and institutional requirements.

## Author contributions

YX: Data curation, Formal analysis, Writing – original draft, Writing – review & editing, Methodology, Visualization, Project administration, Validation. GT: Data curation, Formal analysis, Methodology, Visualization, Writing – review & editing. KT: Data curation, Formal analysis, Methodology, Writing – review & editing. YZ: Data curation, Formal analysis, Methodology, Writing – review & editing. JL: Data curation, Formal analysis, Methodology, Writing – review & editing. WO: Data curation, Formal analysis, Methodology, Writing – review & editing. CS: Data curation, Formal analysis, Methodology, Writing – review & editing. TX: Data curation, Formal analysis, Methodology, Writing – review & editing. CZ: Supervision, Writing – review & editing. YH: Supervision, Writing – review & editing. JJ: Funding acquisition, Project administration, Resources, Supervision, Writing – original draft, Writing – review & editing, Investigation, Visualization.
