# Supplementary material for: Emodin inhibits respiratory syncytial virus entry by interactions with fusion protein
Source: Front Microbiol. 2024 May 16;15:1393511. doi: 10.3389/fmicb.2024.1393511 (PMC11137228; doi:10.3389/fmicb.2024.1393511)
Supplement: Supplementary file 1 [file Image_1.pdf]

Supporting Information

Emodin inhibits respiratory syncytial virus entry by interactions with fusion protein

Yingcai Xiong[a,b*], Guangxing Tan[c*], Keyu Tao[a,b*], Yinghui Zhou[a], Jun Li[b], Weiying Ou[a], Cunsi Shen[a], Tong Xie[a], Chao Zhang[b#], Yayi Hou[d#], Jianjian Ji[a#]

Supporting Figures

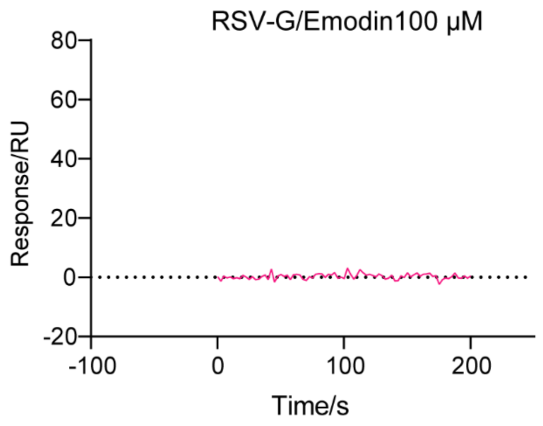

Figure S1. Interaction between RSV-G and Emodin.

We studied the interaction between emodin and RSV-G protein by SPR. First, the RSV-G protein was fixed to the surface of the SPR chip by amino coupling, and then Emodin was injected into the SPR system in PBST buffer containing 1%DMSO at a concentration of 100 uM. Record the change of the response value over time.
